# Cystic schwannoma of the pelvis

**DOI:** 10.1308/003588413X13511609956697

**Published:** 2013-01

**Authors:** T Jindal, S Mukherjee, MR Kamal, RK Sharma, N Ghosh, SN Mandal, AK Das, D Karmakar

**Affiliations:** Calcutta National Medical College,India

**Keywords:** Schwannoma, Pelvis, Laparoscopy

## Abstract

Schwannomas are benign tumours that arise from the Schwann cells of nerve fibres. They commonly occur in the head and neck, mediastinum and extremities. They are extremely rare in the pelvis. These are usually slow growing tumours and are often detected incidentally. Pre-operative diagnosis is extremely difficult as there are no definitive signs on imaging. Aspiration biopsy is often inconclusive or misleading. Surgical excision is both diagnostic and therapeutic. As these tumours are often large in size, open excision is most commonly performed. We describe a case of a large, cystic schwannoma of the pelvis causing bladder outlet obstruction and bilateral hydroureteronephrosis. Complete surgical excision was performed laparoscopically.

Schwannomas are benign tumours that arise from the Schwann cells of nerve fibres. They commonly occur in the head and neck, mediastinum and extremities. They are extremely rare in the pelvis. They are difficult to diagnose as there are no specific clinical or radiological signs and they can mimic a number of pelvic lesions.^1^ These tumours are usually solid but may undergo necrosis and cystic degeneration.^2^ Surgical excision is both diagnostic and therapeutic. As these tumours are often large in size, open excision is most commonly performed. There are only anecdotal reports in the literature where excision of a pelvic schwannoma was performed by minimally invasive techniques.^1,3–5^ We describe a case of a large, cystic schwannoma of the pelvis causing bladder outlet obstruction and bilateral hydroureteronephrosis. Complete surgical excision was performed laparoscopically.

## Case history

A 54-year old man presented to us with history of obstructive urinary symptoms for 2 years. He also complained of gradually progressive distension of the lower abdomen as well as numbness of the left hemiscrotum and medial aspect of the thigh. On examination, there was a 10cm × 8cm mass extending from the umbilicus to the pubic symphysis. The lower margin of the mass could not be palpated per-abdominally. The mass was dull on percussion. On rectal examination, the mass was anterior to the rectum. The prostate could be felt separately. There was no neurological deficit apart from loss of sensation of touch over the femoral triangle and lateral part of the left hemiscrotum. The haematological examination, serum biochemistry and urine analysis were normal.

**Figure 1:**
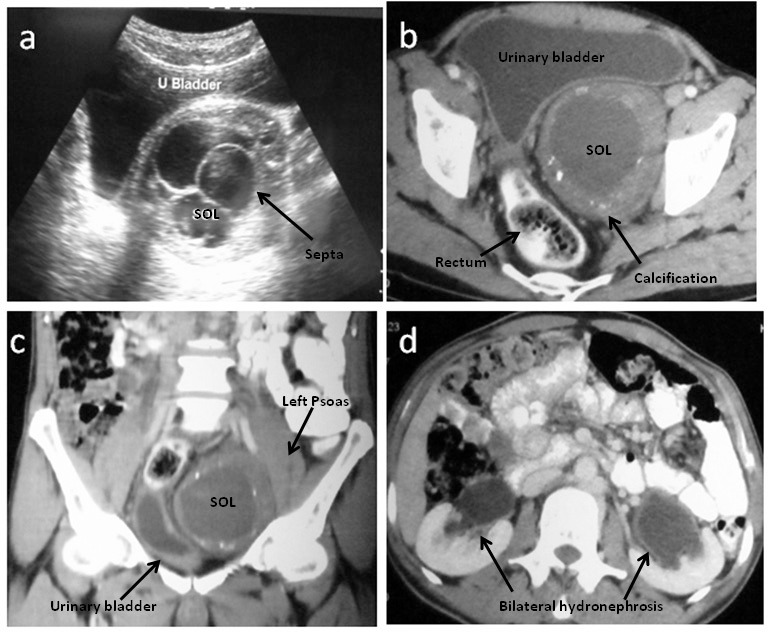
Cystic schwannoma imaging: Ultrasonography of the urinary bladder showed the cystic space occupying lesion (SOL) with multiple septations (a). Computed tomography of the abdomen showed a well defined cystic SOL (11cm × 8cm × 7cm) with multiple areas of focal calcification in the wall. The bladder and the rectum were pushed to the right (b). Coronal sections demonstrated the SOL to be intricately related to the left psoas muscle (c). There was bilateral hydroureteronephrosis (d).

Ultrasonography of the abdomen revealed a cystic mass with thin septations located between the bladder and rectum (Fig 1a). Contrast enhanced computed tomography (CT) of the abdomen revealed a well circumscribed, cystic mass of 11cm × 8cm × 7cm. There were multiple septations and focal areas of calcification in the wall of the mass. The major bulk of the mass was on the left side, and it pushed the bladder and rectum to the right. The mass was intricately related to the left psoas muscle. The planes with the bladder, rectum and the pelvic vasculature were well maintained. There was bilateral hydroureteronephrosis (Figs 1b–d). A diagnosis of pelvic hydatid disease was considered but the serology was negative. An ultrasonography guided biopsy of the mass performed subsequently was inconclusive.

**Figure 2:**
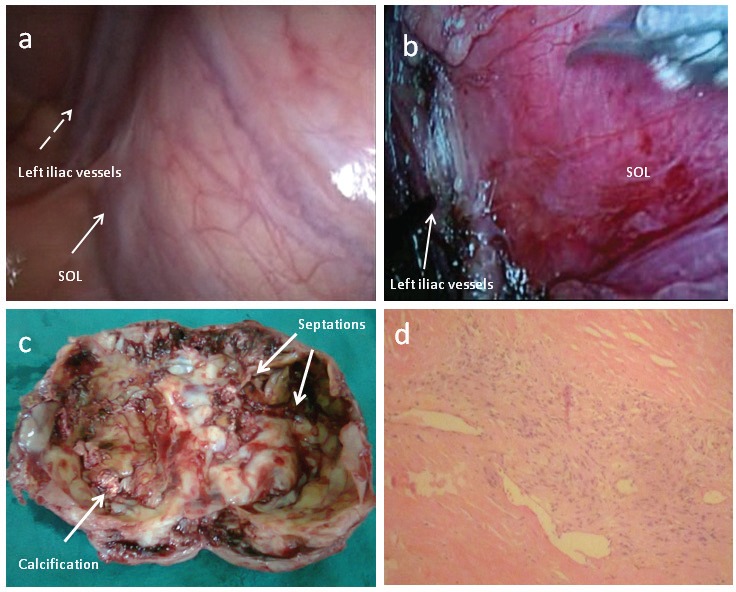
Cystic schwannoma: The mass as seen on laparoscopy (arrow) with the left iliac vessels (dotted arrow) (a). The mass was dissected from the left iliac vessels (b). The gross specimen revealed multiple septations and focal areas of calcification in the wall (c). Microscopic examination (haematoxylin and eosin stain, 10× magnification) revealed the elongated, spindle shaped cells with prominent nuclei arranged in a palisading manner with Antoni A and Antoni B patterns suggestive of a schwannoma (d).

Laparoscopic excision of the mass was planned. Cystoscopy and stenting of the ureters was performed. Four trocars were inserted below the umbilicus in an inverted U fashion and laparoscopy was performed with a 30° scope (Fig 2a). The peritoneum between the mass and the bladder was incised. The plane was developed by blunt and sharp dissection. The peritoneum between the mass and the rectum was then incised and the plane was developed. The ureters were identified and dissected free. The mass was adherent to the left iliac vessels and could only be dissected with difficulty (Fig 2b). There was iatrogenic trauma to the left iliac artery, which was repaired.

The mass was removed following aspiration of the contents. The cut section of the tumour showed a cystic mass with areas of calcification (Fig 2c). The histopathological examination revealed numerous elongated, spindle-shaped cells with prominent nuclei. The cells were arranged in a palisading manner with Antoni A and Antoni B patterns. There were no mitotic figures (Fig 2d). Immunohistochemistry revealed intense staining with S100, confirming the diagnosis of ancient schwannoma.

The patient made an uneventful recovery and was doing well at the six-month follow-up appointment. The numbness of the left hemiscrotum and medial part of the thigh failed to improve, prompting us to presume that the schwannoma originated from the left genitofemoral nerve. The non-visualisation of the left genitofemoral nerve during surgery corroborated this presumption.

## Discussion

Schwannomas are tumours of neurogenic origin. They may arise *de novo* or may be associated with von Recklinghausen’s disease. They are most commonly benign but may undergo malignant changes if they are a part of von Recklinghausen’s disease. They can occur in any part of the body but are extremely rare in the pelvis with fewer than 20 cases reported to date in the English literature.^1^ They are slow-growing tumours and do not cause many symptoms until they have attained a large size. They may cause non-specific symptoms such as backache, abdominal or pelvic heaviness, distension and discomfort.

Pre-operative diagnosis of pelvic schwannomas is difficult as there are no imaging findings that are specific for schwannomas. The differential diagnoses include psoas abscess, adnexal mass, fibrosarcoma, liposarcoma, ganglioneuroma, hydatid cyst and haematoma. CT and magnetic resonance imaging may reveal a well circumscribed mass with areas of calcification, cystic degeneration, haemorrhage and necrosis.^2^ Cystic changes occur as the tumour overgrows its blood supply and such tumours are designated ‘ancient schwannomas’.

As imaging findings are not specific, percutaneous biopsies are often used but they have been reported to be inaccurate and occasionally misleading. Surgical excision and histopathological examination is therefore necessary to reach a confirmatory diagnosis. As these tumours usually attain a large size, open surgical excision is generally performed. There are only anecdotal reports of successful laparoscopic excision of these tumours.^1,3–5^ To the best of our knowledge, this is the first and the largest ancient schwannoma of the genitofemoral nerve that could be excised laparoscopically. As these tumours are most often benign, the prognosis of patients after surgical excision is good.

## Conclusions

Pelvic schwannomas are difficult to diagnose pre-operatively. A high index of clinical suspicion is required. Pre-operative imaging and biopsy are often inconclusive, and surgical excision is the mainstay of treatment. Laparoscopic excision is feasible and should be offered to the patients.
